# Analysis of the Stability of the Body in a Standing Position When Shooting at a Stationary Target—A Randomized Controlled Trial

**DOI:** 10.3390/s22010368

**Published:** 2022-01-04

**Authors:** Marlena Krawczyk-Suszek, Blanka Martowska, Rafał Sapuła

**Affiliations:** 1 Department of Physiotherapy, Medical College, University of Information Technology and Management in Rzeszow, 2 Sucharskiego Str., 35-225 Rzeszów, Poland; bmartowska@wsiz.edu.pl; 2 Zamosc Clinic of Rehabilitation, 22-400 Zamosc, Poland; rafal.sapula@wp.pl

**Keywords:** balance, foot loading, stabilometric parameters

## Abstract

Postural stability of the body depends on many factors. One of them is physical activity. It is especially important in the case of sports or professional work, which combine mobility with the accuracy of a shot in a standing position. The smaller the body fatigue, the more accurate the shot. The aim of the study was the assessment of the impact of physical effort on the center of gravity deflection and length of the COP (center of pressure) path, as well as the reaction of ground forces in people who do not engage in systematic physical activity. The study group included 139 people (23.1 ± 5.2 yr; M: 46.8%; F: 53.2%). The test consisted of performing a static test twice, shooting at the target in a multimedia shooting range. Group X performed the Harvard test between the static tests. Group Y made no effort. The reaction parameters of the ground forces were assessed using the Zebris PDM-L Platform. In Group X performing the Harvard test, an increase in the average COP, VCOP, and 95% confidence ellipse area was noted. The path length and the average velocity of COP speed increased. There were no differences in Group Y (*p* > 0.05). Physical effort significantly affected the postural stability of the studied people, increasing the average parameters assessing balance when adopting static firing position.

## 1. Introduction

The ability to maintain balance, both static and dynamic, depends on the proper functioning of the position control system [1]. Only the correct integration, processing, and coordination of the visual stimuli coming from the periphery, from the balance organ and proprioreceptors, allows the projection of the center of gravity of the body (COG) to be maintained within the quadrilateral of support [2,3]. Postural stability depends on individual physical characteristics, i.e., gender, body weight, muscles, etc. [2].

In order to control body balance and posture, three main sensory systems are involved: vestibular, visual, and proprioceptive [4,5]. The vestibular organ plays a major role in controlling the balance. It is responsible for the control of reflex reactions of the body [6] and the return of the body’s center of gravity to its balance [7,8]. The central nervous system supervises the maintenance of balance by integrating sensory stimuli from the peripheral nervous system and motor signals from the musculoskeletal system [9,10,11]. Postural control is also influenced by genetically determined reactive strategies such as ankle strategies, hip strategies, and steps strategies [12,13].

Support quadrangle is the basis of postural stability and balance, on which the center of gravity falls perpendicularly [14]. These are optimal conditions to maintain a stable upright posture, which is especially important when shooting to a target. In the case of upright posture, the ratio of the length of the foot to the height of a studied person is unfavorable and it is on average in a ratio of 1:7, which is not stability perfection. Moreover, we have visible sexual dysmorphism in the proportions of the feet to the height [15]. In natural conditions, there is no moment of longer body retention in perfect balance. While standing freely, the center of gravity makes small random movements with an amplitude of several millimeters. However, it is an indicator of the proper functioning of the balance control system [16].

Posturography is an objective, accurate, and complex method for assessing balance and load on the feet [9]. With the help of stabilometric platforms, it is possible to analyze the projection of the center of gravity on the base area, in terms of the path length it has traveled, as well as assess the average velocity of the center of gravity and ground forces reactions [17]. Posturographic research platforms are equipped with strain gauges recording the pressure forces and moments of forces exerted on the ground by the respondent’s feet. During the measurements process, while free-standing, changes in the direction of displacement of the projection of the center of the pressure of the feet (COP) onto the platform are registered [18].

Physical effort is one of the factors strongly affecting body stability. We define physical effort as the total kinetic energy required to engage in activity to change the current state to the desired final state [19], i.e., changes in the position of one part of the body in relation to another. Maintaining a stable upright posture is independent of the will of the human being and is a process without conscious participation [20]. The body structure, which consists of several segments connected by the joint-muscular system, ligament system, as well as tendon structures, plays a significant role in this respect [21,22]. Balance control means the static and dynamic balancing of the destabilizing forces of gravity and inertia by stimulating the appropriate muscles groups [23]. The forces generated by muscles compensate for disturbing the balance [24]. Therefore, the parameters of the center of gravity deflection and the reaction of ground forces, after physical exertion with a visible phenomenon of muscle fatigue, differ significantly [25]. Scientific studies have shown that a high level of static balance in shooting disciplines affects high scores [26,27,28]. In biathlon, stability of posture while shooting is crucial for obtaining excellent results in this element [29]. A similar situation occurs in archery, where even slight fluctuations in the center of gravity of the body just before the shot affect accuracy [30].

The mechanism of maintaining an upright “super-stability” position of shooters, more often than in other sports, probably relates to their strong motor ability to “freeze” while shooting [31,32,33]. Hence the choice of a shot at a stationary target, in the research presented below, as the activity most strongly correlated with the need to maintain an upright position with the smallest possible amplitude of deviations of the center of gravity. As far as shooting disciplines are concerned, there is a confirmed correlation between stabilometric parameters and the level of performance, for this reason, the assessment of stability with determination of ground force response can be considered a conventional standard physiological test and a test of the level of mobility while maintaining a standing posture [27,31]. With reference to such sports as biathlon or military activities, shooting is a basic element in the cycle of physical activities [27,34,35,36]. The literature reports that postural stability increases during dual-tasking tests, when in addition to maintaining a stable upright position, the respondent’s needs to concentrate on another cognitive task [37,38]. The research results indicate that the concentration process can facilitate the posture static control [39]. For this reason, the choice of trying to shoot a stationary target in a standing position after exercise is justified. There was little research on body stability when shooting based strictly on a group that does not conduct regular physical exertion and exercises to improve body stability. Both factors strongly correlate with the deflection amplitude [40]. The more trained the competitor is, the more experienced the shooter is, the body deflects less, the better the stability and accuracy of the shots are [27,36,41]. Therefore, in the first step, it is necessary to check the path followed by COP in people without physical and shooting training, in order to be able to assess people with shooting experience and trained in subsequent tests and make a proper comparison. Pedobarography is commonly used in many diseases, while the application of pedobarography in sport is rare [17].

The aim of the study was the assessment of the impact of physical effort on the center of gravity deflection and length of the COP path, as well as the reaction of ground forces in people who do not engage in systematic physical activity with the application of a shot to a stationary target. The aim of the research was to indicate significantly higher parameters in group X after the Harvard test, which would confirm the occurrence of a higher amplitude of body deflections after physical exertion.

## 2. Materials and Methods

### 2.1. Study Group

The study group included 139 people (M: 65, 46.8%; F: 74, 53.2%). Group X involved 45.7% women and 54.3% men, Group Y—60.9% of women and 39.1% of men. Gender did not significantly differentiate the X and Y groups (*p* = 0.072). The average age of the studied people was 23.1 ± 5.2 years old. The mean age in group X was 23.4 ± 5.6, while in group Y it was 22.8 ± 4.5 years old. There were no significant age differences between the compared groups (*p* = 0.746). A place of residence was also not a differentiating factor (*p* = 580). The compared groups differed significantly in terms of education. However, the limitation involved a low number of people with higher (N = 3) and secondary (N = 31) education. (Table 1) The reason for such a low number of people with higher education was the age of the respondents, most of whom did not graduate. All the studied people were right-handed. The respondents were informed about the anonymity and voluntariness of the tests and about the possibility of resignation at any stage. The respondents declared no contraindications to physical effort. Each respondent provided his/her written consent to participate in the research. The exclusion condition was a visual impairment, neurological diseases, including neuropathy, diseases of the musculoskeletal system that make it difficult to perform a task, disorders of the labyrinthine function, middle ear diseases with symptoms of vertigo, and problems with maintaining body balance associated with other diseases. None of the respondents declared previous experience in shooting weapons at a multimedia shooting range, or competitive sports. 

**Figure 1:**
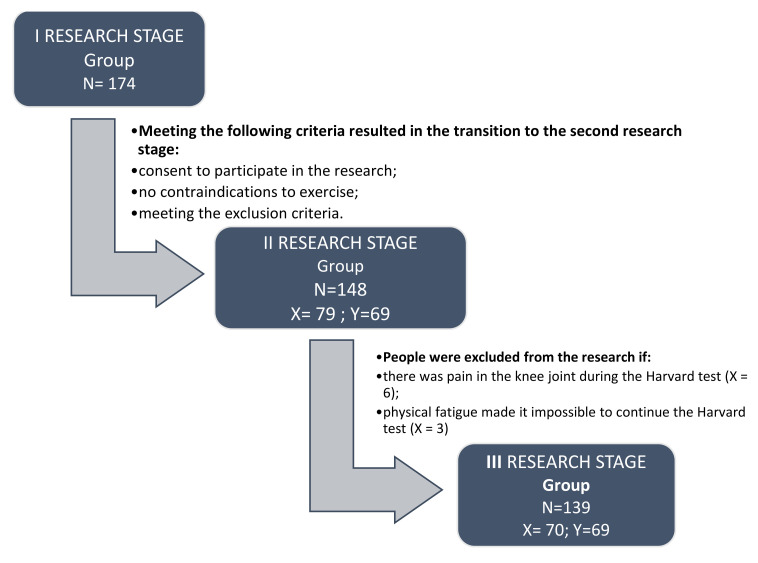
Study flow diagram.

A total of 174 people were recruited for research in the first stage, while 148 people were included in the second stage because they met all the inclusion and exclusion criteria. Qualification for groups X and Y of people recruited to the second stage was random. A stratified sampling was performed in the database (N = 148) to ensure the representativeness of both compared groups. The qualification was carried out in the second stage of the research. The actual research was completed by 70 people qualified to group X and 69 people qualified to group Y. The number of excluded people and the flow of people are presented in detail in Figure 1.

The study was performed in accordance with the Helsinki Declaration (WMA Declaration of Helsinki—Ethical Principles for Medical Research Involving Human Subjects) [42].

### 2.2. Methodology of the Study

The study was based on a static test on the stabilometric Zebris PDM-L platform, made by the German company, Zebris Medical GmbH, with dimensions of 1370 mm × 535 mm × 15 mm (l × b × h). The size of the measurement area is 1220 mm × 474 mm (l × b). It allowed assessing the reaction of the ground forces and the deviations of the center of gravity of the respondents. The Zebris PDM-L platform is made of a matrix containing 8064 individually calibrated, capacitive force sensors that enable the analysis of forces under the feet while standing and walking. In addition, the parameters of the platform allow an accuracy of ±5% (FS) in the measuring range of 120 N/cm^2^, with a sampling frequency of 120 Hz [43].

The test included standing the foot without footwear and shooting to a stationary target facing the studied person (SS). The test duration was 30 s.

The study included a multimedia shooting range and GLOCK (replica) type short weapons of dimensions (l/h/b): 202/139/34 mm and weight 710 g, not posing a threat to the studied people. Before the first actual test, each of the subjects was instructed to shoot and attempted to shoot to a stationary target. The test did not exceed 15 s. Each of the studied people was provided with the same conditions to learn about the weapon and the shooting method. The shot was fired in the position of standing on both feet and extending both upper limbs towards the target-the dominant limb, the limb holding the weapon, the other limb, stabilized during the shot. The repeatability of foot positioning on the Zebris PDM-L platform, thus ensuring the repeatability of setting the distance of the studied person from the screen of the multimedia shooting range, was conditioned by the lines permanently adhesive on the platform and marking the center line, which coincided during subsequent static tests with the center line of the body of the studied person. The studied people stood on the platform, facing the surface of the multimedia shooting range, with a panel on the right side of the body indicating the waking state of the Zebris PDM-L platform.

The center line was determined in relation to the platform field, which indicated the load parameters by actively recording the body pressure forces on the ground. Field dimensions: 124 × 53 cm. The marked center line exactly coincided with the center line of the indicated field actively registering ground reaction.

The distance between the line determining the repeatability of the foot placement and the outer edge of the platform [was closer to the marked line] was 15.5 cm, while the distance between the same line and the shooting range screen was 5 m.

The study was performed in two groups. After the first static test with shooting (ISS—static test with shooting before effort), the first study group (Group X) performed the effort test, i.e., the Harvard test. The test involved climbing a 50 cm high step at a rate of 30 steps per minute, for the next 5 min. In order to maintain the same conditions to carry out the test, the pace of climbing the step was measured by a metronome. Immediately after the end of the Harvard test, the studied people performed the same static test with shooting (IISS) while maintaining the methodology and conditions the same as those in the first pre-exercise test (ISS). The time to start the second test (IISS) after the end of the Harvard test was 10 s (time to go between the step and the Zebris PDM-L platform and the correct positioning of the respondent on the platform to carry out the second test).

The control group (Group Y) did not perform the Harvard test. The studied people successively performed two static tests with shooting (ISS and IISS), between which they rested for 5 min without undue physical effort, while maintaining the methodology and conditions the same as those performed for Group X.

**Figure 2:**
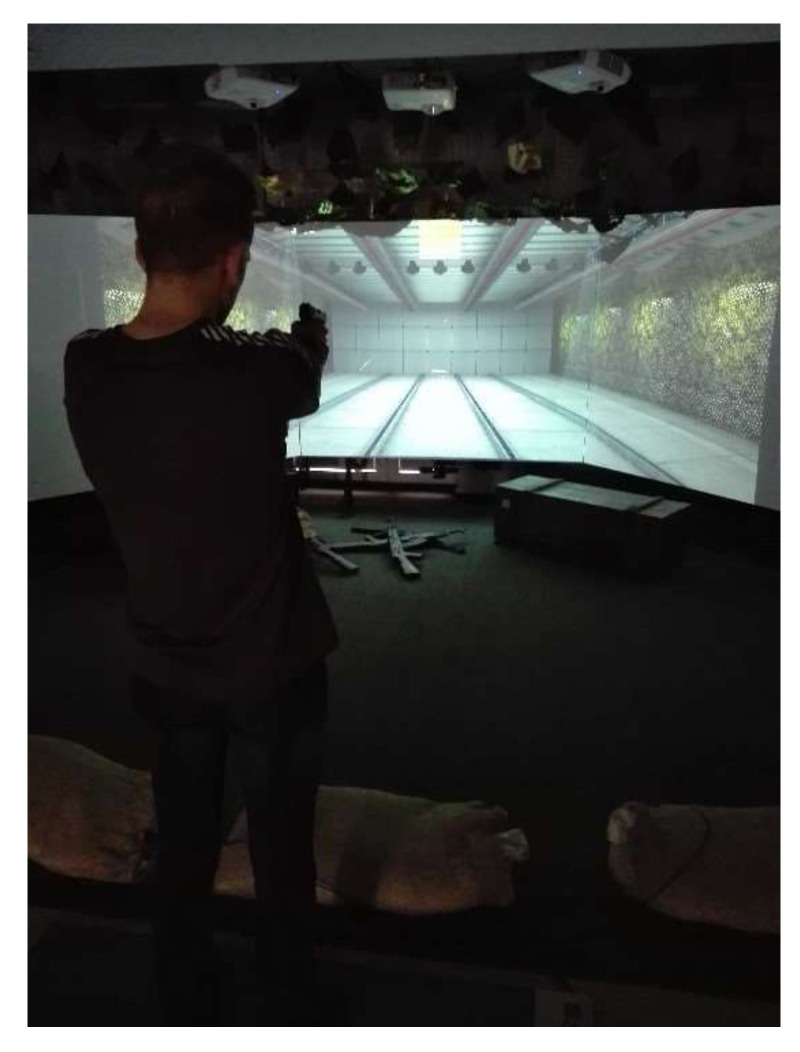
Stay at Platform Zebris PDM-L and shooting at the target.

The analyzed parameters are as follows:

– 95% confidence ellipse area [mm^2^]—defines the size of the area marked by the COP; ellipse area includes 95% of the COP measurement points; this parameter makes it possible to assess the size of the area of the COP movement on the support surface.
– COP path length, [mm]—defines the total length of the path marked by the COP; the sum of distances between the locations of the COP constitutes the path length.
– COP average velocity [mm/s]—defines the mean velocity at which the COP moves; this parameter indicates the speed of changes in the COP location, which reflects the speed of postural reactions while standing.
– Forefoot force L [%]—describes the reaction of the ground forces for the forefoot of the left limb.
– Backfoot force L [%]—describes the reaction of the ground forces for the rear-foot left limb.
– Total force L [%]—indicates the overall load on the left limb.
– Forefoot force R [%]—describes the reaction of ground forces for the forefoot of the right limb.
– Backfoot force R [%]—escribes the reaction of ground forces for the rear-foot right limb.
– Total force R [%]—indicates the overall load on the right limb [43,44] (Figure 2).

The research was conducted in March–June 2021.

### 2.3. Statistical Analysis

The analyzed parameters did not have a normal distribution (W Shapiro-Wilk test <0.05). In the analysis, the Mann–Whitney U was used test to compare significant relationships between groups of independent variables and the Wilcoxon test for analysis of dependent measurements from sample I and sample II in a given group. Linear comparison of qualitative variables was performed using Pearson’s Chi-square test. Purpose of the assessment correlation between the shooting result and individual parameters, logistic regression was carried out. The power of the test was assessed with the hypothesis: Mi1 = Mi2 and the sample size N1 = 70 and N2 = 69 The power of the test is 1. There were no missing data. Parameter values assessed with the use of the Zebris PDM-L platform were fully generated in the reports of the respondents from the successively carried-out static tests on the platform. The ZEBRIS PDL platform works with the WinFDM Zebris version 1.16.12 software from Zebris GmbH-Germany production, which enables the reading of the parameters of the ground force reaction and generating reports from statistical and dynamic tests performed on the Zebris platform.

Statistical dependences were significant if their level of significance was *p* < 0.05. The analysis was carried out using Statistica 13.0 PL.

## 3. Results

The analysis of individual parameters of the center of gravity deflection and the distribution of relative forces of the ground during the static test with shooting was assessed for the two compared groups. In the first test (ISS), significant differences were noted in the COP path length (*p* = 0.009) and VCOP average velocity (*p* = 0.009). Both parameters were higher in group X. Differences were also indicated in the forefoot and backfoot of the right limb (*p* = 0.007). A higher percentage response of ground forces was noted in the right forefoot of people in group Y (56.4 ± 21.5%) compared to group X (47.8 ± 16.8). The maximum recorded load in both groups was 100%. A proportionally inverse value was recorded for the right backfoot. The average load for group X was 52.2 ± 16.8%, while in group Y this value was lower and amounted to 43.6 ± 21.5%. The maximum value of backfoot force R in group X was 83%, while in group Y was 93%. The differences in the load on the left foot in each of the analyzed parameters in both compared groups were insignificant. The analysis did not confirm any significant differences in the compared groups (*p* > 0.05).

The parameters that significantly differed in the two compared groups were, in particular, 95% confidence ellipse area, COP path length, and VCOP average velocity (*p* < 0.001). The average of these parameters were higher in group X, which performed physical effort between two consecutive static tests. This indicates the impact of effort on extending the average speed of the center of gravity of the studied people and the distance they traveled.

The average parameters recorded in two consecutive tests for groups X and Y were analyzed. Statistically significant relationships were indicated in the mean values of parameters in group X, where the 5-min Harvard test was performed. The center of gravity deflection parameters, i.e.: COP path length, VCOP average velocity, and 95% confidence ellipse area increased after physical effort. The differences in the measurements in the first and second tests were statistically significant in all cases (*p* < 0.001). The path length increased from 340.9 ± 181.4 mm to 447.3 ± 211.0 mm. The VCOP average velocity increased from 11.9 ± 6.4 to 15.7 ± 7.4 mm/s (Table 2).

Significant changes were also noted in the parameters of the distribution of relative ground forces, both in the case of the forefoot and backfoot, as well as the right and left lower limbs (*p* < 0.001). In the first tests, a higher index of the percentage distribution of ground forces was recorded in the backfoot, while after the Harvard Test, the higher load was transferred to the forefoot. In the analysis of the reaction of the ground forces after the effort, significant differences were found between the forefoot and backfoot forces. In the lower-left limb, the difference in mean measurement was 4.8%. However, the difference in the mean measurement in the right limb, where the forefoot force was 56.5% and the backfoot 43.5%, was as much as 13%. While in the first test, the difference for the same limb was only 4.4%. Increasing the load on the forefoot of the right lower limb is a consequence of the right-handedness of the studied people and the need to stabilize the body after physical exertion in order to shoot as correctly as possible to the target.

There were no significant differences between the average parameter measurements in two consecutive tests in group Y (*p* > 0.05). However, it is worth emphasizing that in this group of people, the average path length and VCOP average velocity were lower in the second test than in the first one (Table 3).

The correlation between the number of points obtained in two 30-s tests at the multimedia shooting range and individual stability parameters was analyzed. In the control group Y, no statistically significant relationships were found between the analyzed variables. However, in group X, where the Harvard test was carried out, only in the case of the parameter: 95% confidence ellipse area and backfoot force R significant relationships were found. There was a positive correlation between the number of points in the first test (ISS) and the parameter 95% confidence ellipse area. However, in the second test (IISS), the correlation was negative (r = −0.28). There was a negative correlation between the number of points obtained and the backfoot force R parameter (r = −0.25). Other analyzed correlations between the number of points obtained and the parameter values in group X did not show any significant differences in the ISS and IISS measurements (Table 4).

## 4. Discussion

In the ISS test, the mean COP path length and VCOP average velocity values were significantly higher in group X compared to group Y. The mean load of forefoot force R was significantly higher in group Y, while backfoot force R was significantly higher in group X. In the IISS test, the parameter values were almost twice as high: 95% confidence ellipse area, COP path length, and VCOP average velocity was recorded in group X after the Harvard test, compared to group Y, which did not do it. The value of other assessed parameters did not differ significantly between the compared groups. When analyzing the parameter values in the measurement of ISS and IISS, significant differences were noted in group X. However, in group Y, no significant changes were noted between the measurements: ISS and IISS. In the case of the correlation of the number of points obtained from shooting with the analyzed parameters, only in group X there was a significant correlation in the ISS and IISS measurement in 95% confidence ellipse area and in the IISS measurement in backfoot force R.

The balance control process is based on stimuli coming from the labyrinth, vision, proprioceptors, and tactile receptors [45,46]. All pathological or functional changes that impair the functioning of the postural control system or the executive system are reflected in disturbances of posture balance and stability [47,48]. Dysfunctions of the sense organs and deep sensation disturb measurements of body balance [49]. In our own research, the exclusion criteria involved vision defects and problems with maintaining a balance of the body correlated with the aforementioned diseases, which allowed us to minimize the factors disturbing the reading of the balance parameters analyzed on the Zebris PDM-L platform.

When shooting to a target, body stability is an important indicator of the effectiveness and accuracy of shots [27,31,50]. This relationship can also be considered with various sports, i.e., basketball, biathlon, wherever the sudden stop of the body after exercise requires stabilization of shooting, throwing, etc. to a target [26,51]. The balance mechanism, on the other hand, consists of equalizing forces and moments acting per unit of time on the body—sportsmen/women must maintain balance despite destabilizing factors [52,53]. An important element affecting the center of gravity deflection is the upper limbs, which are perpendicular to the torso when shooting. Shooting position is an unnatural position of the body forcing isometric muscle tension [54]. The authors studied the effect of the body posture stability on the results [27,33,50,55], as well as compared the posture stability of shooters, swordsmen/women, and control groups under different conditions. In each analysis, shooters took the most stable body posture [56]. However, in the author’s study, the study group consisted of people who did not have professional experience in sport and shooting. Upright and stable posture after exercise requires more muscle work. The studies showed that the foot load changed. After the Harvard test, a higher load was recorded for the forefoot, in particular for the right lower limb (*p* < 0.001). The difference between the load of the right lower limb in terms of forefoot and rearfoot after exercise differed by 13%, where in the first test, it was only by 4.4%. Physiological conditions of the body posture indicate an even load on both limbs, as well as the forefoot and rear-foot. The literature studies report that in the group of shooters, a slight predominance of average parameters of ground reaction forces was noted, indicating 53.5% of average rear-foot load [54]. This load distribution is appropriate for a stable body posture when shooting, without exercising before the test, which is one of the elements of the shooting technique. Similar relationships are indicated by Sadowska et al. confirming the impact of the posture stability [57]. Other results indicate that in subsequent static shooting tests performed at distances every 2 km during the run, there were no significant differences in the parameters of the body stability of the studied people [58]. In the author’s study, physical effort by moving the body’s center of gravity throws the body out of balance and determines the change in load from rear-foot to forefoot. In conditions of fatigue, the nervous system is unable to compensate for disturbances in the functions of the body’s stabilizing system, so balance and balanced posture cannot be achieved by humans [59].

The study noted an increase in the center of gravity (VCOP) speed and path length after exercise. The increased speed of VCOP after a series of runs is confirmed by Sadowska et al., and studies by other authors using treadmill running [60], moderate running track [61], or in the case of long-lasting treadmill exercises conducted at subsequent oxygen and anaerobic thresholds [62].

In the control group, where no effort was made, no significant difference was noted in the parameters listed in two subsequent measurements (Group Y). However, a decrease in the average VCOP speed from 9.6 to 8.6 m/s was observed, as well as a shortening of the COP path from 273.5 to 254.7mm, which can mean that muscle learning allowed the studied people to better stabilize their posture in the second test of shooting to a target.

The author’s own research assessed the effect of exercise on maintaining a stable standing posture while firing a shot at the target. Muscle fatigue is an exercise-induced reduction in a muscle’s ability to generate strength or power. This definition is used to describe a temporary decline in the ability to perform physical activities [63,64]. As reported in the literature, muscle fatigue reduces postural stability, regardless of the tired muscle area, and the asymmetry of the load on the lower limbs increases [65,66].

By analyzing the ability to control balance based on 95% confidence ellipse area [mm^2^], it was confirmed more than doubling the field in group X, where the Harvard test was performed. Analyzing the relationship between the studied groups in the second measurement after exercise, a significantly more than twice higher parameter 95% confidence ellipse area [mm^2^] in the X group (441.9 mm^2^) compared to the Y group (201.7 mm^2^) (*p* < 0.001) was noted. This parameter confirms the decrease in the ability to control the balance parameters after physical exercise [67].

Balance, both in the group of patients, healthy people and in the group of sportsmen/women is an important determinant of the correct movements. Stability affects sports performance, accuracy, and precision, which is confirmed by many available studies [30,50,56,68,69,70,71,72,73,74]. Balance parameters of sportsmen/women in a group of people who lost their motor function can be correctly affected by long-term training [51,73,75]. 

The presented correlation of the obtained scores while shooting to individual balance parameters in the group of untrained people, without shooting experience, did not confirm a significant relationship between COP deflections in both standing posture and the level of shooting results. The same conclusions, but relating to sports groups, can be found in the literature [76,77,78,79].

The Zebris platform for assessing the ground force reaction is a perfect tool for verifying the stability of the body in both static and dynamic tests [18,80,81,82,83]. Research on postural stability disorders using the Zebris platform is widely used in the clinical assessment of patients at risk of falling [84,85,86,87].

The aging process significantly affects posture efficiency due to age-related changes in sensor-motor and cognitive functions [48]. Early detection of abnormalities in the balance control allows for appropriate preventive interventions, e.g., rehabilitation, which is of key importance in reducing the risk of falls [86,87]. The assessment of balance using clinical tests is subjective and is not sensitive enough to identify early postural control disorders [9,85]. Clinical assessment of balance is most often able to detect abnormalities only when significant pathological changes have already occurred in the body posture control system [9]. The data provided with this method are qualitative, not quantitative. The use of standardized tests and clinical scales such as the Berg Balance Scale or the Tinetti Test is time-consuming [85,88,89]. Instrumental methods, such as posturography, are an alternative to clinical assessment. These are objective and sensitive methods for assessing balance, which allows early recognition of subtle abnormalities in postural control, based on the analysis of foot loading parameters and their deviations from the normative values [9,80,90]. Tests using the PDM-L Zebris platform provide accurate and comprehensive measurements of balance parameters. Calibration of the platform’s sensors before each measurement ensures the reliability and credibility of reporting stabilometric parameters.

For the assessment of postural stability, it is important to analyze parameters such as the length of the COP path, the path speed (VCOP), the area of the ellipse defined by the COP movements, and the symmetry of the limb loading [21]. Hence, many researchers analyze these parameters when assessing static balance [18,80,84,85,91,92,93,94,95]. The own research also confirmed a significant impact of exercise on the following balance parameters: 95% confidence ellipse area, COP path length, and VOP average velocity. The value of these parameters was almost twice as high in the group of people who took the Harvard test compared to the second group. The use of the Zebris PDM-L platform and its software allow for the analysis of these parameters and the comparison of the results obtained by the tested subjects with the normative values specified for the appropriate age range. During free-standing, the position of the COP changes slightly, while maintaining a stable body position. In this way, an area is created within which the COP operates―the so-called ellipse area. In the case of stabilometric tests, the assessment of parameters such as COP is inversely proportional to the value of the feature. This means that the more effective the posture control mechanisms are, the shorter the COP path length and the smaller the ellipse area are [21,91,92].

The analysis of equilibrium parameters enables the assessment of postural stability during sports training monitoring or for diagnostic purposes in the rehabilitation of people with musculoskeletal dysfunctions [80,85,96,97]. In addition, they allow determining the risk of sports-related injuries such as overload injuries, joint sprains, and ligaments injuries [98,99,100]. By assessing the distribution of loads, the platform enables a precise assessment of the shape of the foot arches, which is extremely important in diagnostics and treatment of foot defects [101]. In addition, the Zebris platforms are applied to monitor the process of treatment and rehabilitation of patients with orthopedic injuries [102,103,104,105].

**Limitation.** The presented research results should be viewed with certain limitations. The limitation is the body’s level of fitness and the exercise tolerance of each respondent. Despite the lack of systematic physical activity by the respondents, strictly defined conditions of the Harvard test in the research and compliance with the exclusion criteria, each person has an individual tolerance of physical effort. The result involves a different subjectively perceived level of fatigue for each person, which can affect postural stability and increase the deflection of the center of gravity, which the authors did not involve. In addition, the parameters can be influenced by the weight of a weapon, relative length of an upper limb, which can affect the moment of the shoulder joint (downward direction), as well as gender and age of the respondents. There is no motor memory in untrained people. The height of the center of gravity is also undoubtedly important in maintaining a stable posture.

## 5. Conclusions

Physical effort affects postural stability. Under the influence of physical effort, the center of gravity set in motion, when the body stops, moves a longer path and a higher COP speed is noted compared to the control group, in which no physical effort was performed. Accuracy of shooting does not significantly affect the parameters of the deflection of the body. The research should include other factors for analysis such as muscle mass, arm length etc. as well as a group of experienced and trained people in the shooting discipline to assess the impact of these factors on the stability of posture when shooting at a stationary target. The described results can be used for comparison with the group of the respondents who, in addition to the assessment of postural stability, will be engaged in another cognitive task on the platform. In addition, the obtained research results can be useful in planning sports training programs aimed at reducing the impact of fatigue on the asymmetry of limb load and improving the static balance.

Using the diagnostic capabilities of the platform in the static and dynamic test, it is planned to extend the research on the impact of physical effort on the gait parameters in the dynamic test.

## Figures and Tables

**Table 1:** General characteristics of the research group of the respondents.

Variable	Group				Total		*p*
	X		Y				
	n	%	n	%	n	%	
**Gender:** - female/male	32/38	45.7/54.3	42/27	60.9/39.1	74/65	53.2/46.8	0.072 *
**Place of residence:** - city/village	40/30	57.1/42.9	42/26	60.9/37.7	82/56	59.0	0.580 *
**Education:** - higher/secondary/elementary	3/23/44	4.3/32.9/62.9	2/8/59	2.9/11.5/85.5	5/31/103	3.6/22.3/74.1	0.009 *
**AGE**	**ẋ**	**SD**	**ẋ**	**SD**	**ẋ**	**SD**	
	23.4	5.6	22.8	4.5	23.1	5.2	0.746 **

n—number of observations; %—percent; ẋ—average; SD—standard deviation; *p* *—level of statistical significance Pearson’s Chi-square test; *p* **—level of statistical significance Mann–Whitney U Test.

**Table 2:** Average parameters of ground force reaction in the static test with shooting in individual test groups. Value analysis in two measurements for two compared groups.

Parameters [Unit of Measure]	Group								*p*
	X [n = 70]				Y [n = 69]				
Trial I (ISS)	ẋ	SD	Me	Min–Max	ẋ	SD	Me	Min–Max	
95% confidence ellipse area [mm^2^]	204.2	135.2	165.3	57.4–746	193.3	202.8	131.9	22.5–1117.0	0.062
COP path length [mm]	340.9	181.4	283.5	126.0–964.0	273.5	146.0	228.5	102.5–755.0	0.009
VCOP average velocity [mm/s]	11.9	6.4	9.9	4.4–34.0	9.6	5.1	8.0	3.6–26.0	0.009
Forefoot force L [%]	46.6	15.9	46.1	18.3–100.0	51.1	21.2	47.0	6.4–100.0	0.295
Backfoot force L [%]	53.4	15.9	53.9	0.0–82.0	48.9	21.2	53.0	0.0–94.0	0.295
Total force L [%]	53.0	10.2	51.7	28.5–100.0	52.7	12.0	51.2	22.2–98.0	0.935
Forefoot force R [%]	47.8	16.8	45.0	17.2–100.0	56.4	21.5	52.2	6.7–100.0	0.007
Backfoot force R [%]	52.2	16.8	55.0	0.0–83.0	43.6	21.5	47.8	0.0–93.0	0.007
Total force R [%]	47.0	10.2	48.3	0.2–72.0	47.3	12.0	48.8	1.6–78.0	0.935
**Trial II (IISS)**	**ẋ**	**SD**	**Me**	**Min–Max**	**ẋ**	**SD**	**Me**	**Min–Max**	***p***
95% confidence ellipse area [mm^2^]	441.9	445.4	247.3	60.6–2394.0	201.7	141.2	150.3	32.3–582.0	<0.001
COP path length [mm]	447.3	211.0	390.7	197.2–1200.0	254.7	126.9	235.5	91.2–771	<0.001
VCOP average velocity [mm/s]	15.7	7.4	13.7	6.9–42.0	8.9	4.4	8.3	3.2–27.0	<0.001
Forefoot force L [%]	52.4	20.0	48.9	6.3–100.0	51.0	20.1	47.6	24.9–100.0	0.396
Backfoot force L [%]	47.6	20.0	51.1	0.0–94.0	49.0	20.1	52.4	0.0–75.0	0.396
Total force L [%]	52.8	6.9	51.9	35.8–82.0	51.9	9.4	51.5	16.5–93.0	0.788
Forefoot force R [%]	56.5	20.2	52.5	16.9–100.0	54.8	19.4	48.1	11.5–100.0	0.543
Backfoot force R [%]	43.5	20.2	47.5	0.0–83.0	45.2	19.4	51.9	0.0–89.0	0.543
Total force R [%]	47.2	6.9	48.1	17.9–64.0	48.1	9.4	48.5	6.9–83.0	0.788

ẋ—average; SD—standard deviation; Me—median; Min–Max—reference minimum to maximum; *p*—level of statistical significance Mann-Whitney U Test.

**Table 3:** Influence of physical effort on average parameters of ground reaction in static test with shooting in individual groups. Value analysis for two measurements in each of the analyzed groups.

Parameters [unit of Measure] ISS vs. IISS		Group					
		X [n = 70]		*p*	Y [n = 69]		*p*
		M	SD		M	SD	
95% confidence ellipse area [mm^2^]	I	204.2	135.2	<0.001	193.3	202.8	0.807
	II	441.9	445.4		201.7	141.2	
COP path length [mm]	I	340.9	181.4	<0.001	273.5	146.0	0.820
	II	447.3	211.0		254.7	126.9	
VCOP average velocity [mm/s]	I	11.9	6.4	<0.001	9.6	5.1	0.820
	II	15.7	7.4		8.9	4.4	
Forefoot force L [%]	I	46.6	15.9	<0.001	51.1	21.2	0.387
	II	52.4	20.0		51.0	20.1	
Backfoot force L [%]	I	53.4	15.9	<0.001	48.9	21.2	0.387
	II	47.6	20.0		49.0	20.1	
Total force L [%]	I	53.0	10.2	0.411	52.7	12.0	0.630
	II	52.8	6.9		51.9	9.4	
Forefoot force R [%]	I	47.8	16.8	<0.001	56.4	21.5	0.158
	II	56.5	20.2		54.8	19.4	
Backfoot force R [%]	I	52.2	16.8	<0.001	43.6	21.5	0.158
	II	43.5	20.2		45.2	19.4	
Total force R [%]	I	47.0	10.2	0.411	47.3	12.0	0.630
	II	47.2	6.9		48.1	9.4	

ẋ—average; SD—standard deviation; *p*—level of statistical significance test Wilcoxon.

**Table 4:** Correlation of the number of points obtained in shooting with the stability parameters in individual tests. Value analysis for two measurements in each of the analyzed groups.

Parameters [unit of Measure] vs. Points for Shooting in ISS and IISS		Group			
		X [n = 70] ** 12.8 ± 3.3 (I) 14.2 ± 5.6 (II)		Y [n = 69] ** 13.6 ± 4.4 (I) 13.6 ± 4.1 (II)	
		r	*p*	r	*p*
95% confidence ellipse area [mm^2^]	I	0.34	0.004	0.02	0.869
	II	−0.28	0.018	−0.12	0.359
COP path length [mm]	I	−0.06	0.634	0.21	0.087
	II	−0.07	0.580	0.05	0.697
VCOP average velocity [mm/s]	I	−0.06	0.632	0.21	0.089
	II	−0.07	0.580	0.05	0.696
Forefoot force L [%]	I	0.002	0.990	0.08	0.522
	II	0.21	0.077	0.01	0.939
Backfoot force L [%]	I	−0.002	0.990	−0.08	0.522
	II	−0.21	0.077	−0.01	0.939
Total force L [%]	I	−0.07	0.536	−0.14	0.251
	II	−0.16	0.188	−0.01	0.936
Forefoot force R [%]	I	−0.04	0.761	0.06	0.611
	II	0.25	0.039	0.04	0.775
Backfoot force R [%]	I	0.04	0.761	−0.06	0.612
	II	−0.25	0.039	−0.04	0.775
Total force R [%]	I	0.07	0.536	0.14	0.251
	II	0.16	0.188	0.01	0.936

r—correlation between variable, *p*—level of statistical significance of regression; ** ẋ ± SD—average ± standard deviation of a number of shooting in an attempt ISS(I) and IISS(II).

## Data Availability

Participants data, the full dataset and statistical code are available from the corresponding author (m.krawczyk.umlub@gmail.com). Participant consent for data sharing was not obtained but the presented data are anonymised and risk of identification is extremely low.
